# Update on Pediatric asthma management: Recent advances

**DOI:** 10.1111/pai.70480

**Published:** 2026-09-24

**Authors:** Nitin Dhochak, Polly F. M. Robinson, Sejal Saglani

**Affiliations:** ^1^ Great Ormond Street Hospital London UK; ^2^ Royal Brompton Hospital London UK; ^3^ Evelina London Children's Hospital St Thomas' Hospital London UK; ^4^ National Heart & Lung Institute, Imperial College London & Imperial Centre for Paediatrics & Child Health London UK

**Keywords:** asthma, biological products, biomarkers, disease management, pediatric

## Abstract

Asthma remains the commonest chronic pediatric respiratory disease and a major cause of childhood morbidity worldwide. Recent advances in pediatric asthma management shift the focus from symptom relief alone towards early control of airway inflammation, exacerbation prevention, and personalizing biomarkers to target biologics. This review highlights the growing role of anti‐inflammatory reliever [AIR] therapy and maintenance‐and‐reliever therapy [MART], both of which incorporate inhaled corticosteroids [ICS] with bronchodilator treatment to reduce reliance on short‐acting β_2_‐agonists [SABA]. Pediatric clinical trials demonstrate that strategies including AIR only and MART, compared to SABA reliever, reduce acute asthma attack burden in some disease subgroups while further large trials are underway. For severe pediatric asthma, advances in understanding type 2 airway inflammation have led to the introduction of biologics targeting key inflammatory pathways, including IgE, interleukin (IL)‐5, IL‐5 receptor, IL‐4/IL‐13 signaling, and thymic stromal lymphopoietin. Adult trials have shown reductions in asthma attacks with all currently licensed biologics. Although pediatric evidence remains more limited than in adults, we summarize the current evidence of efficacy in childhood severe asthma. Biomarker‐guided selection using blood eosinophils, fractional exhaled nitric oxide, and serum IgE is increasingly important, yet optimal pediatric biomarkers of response remain uncertain. Current challenges include gaps in child‐specific biomarker validation and uncertainty regarding treatment duration and discontinuation strategies. Overall, contemporary pediatric asthma management is moving towards precision medicine, integrating anti‐inflammatory reliever strategies and targeted biologics to improve disease control, minimize exacerbations, and preserve long‐term lung health.

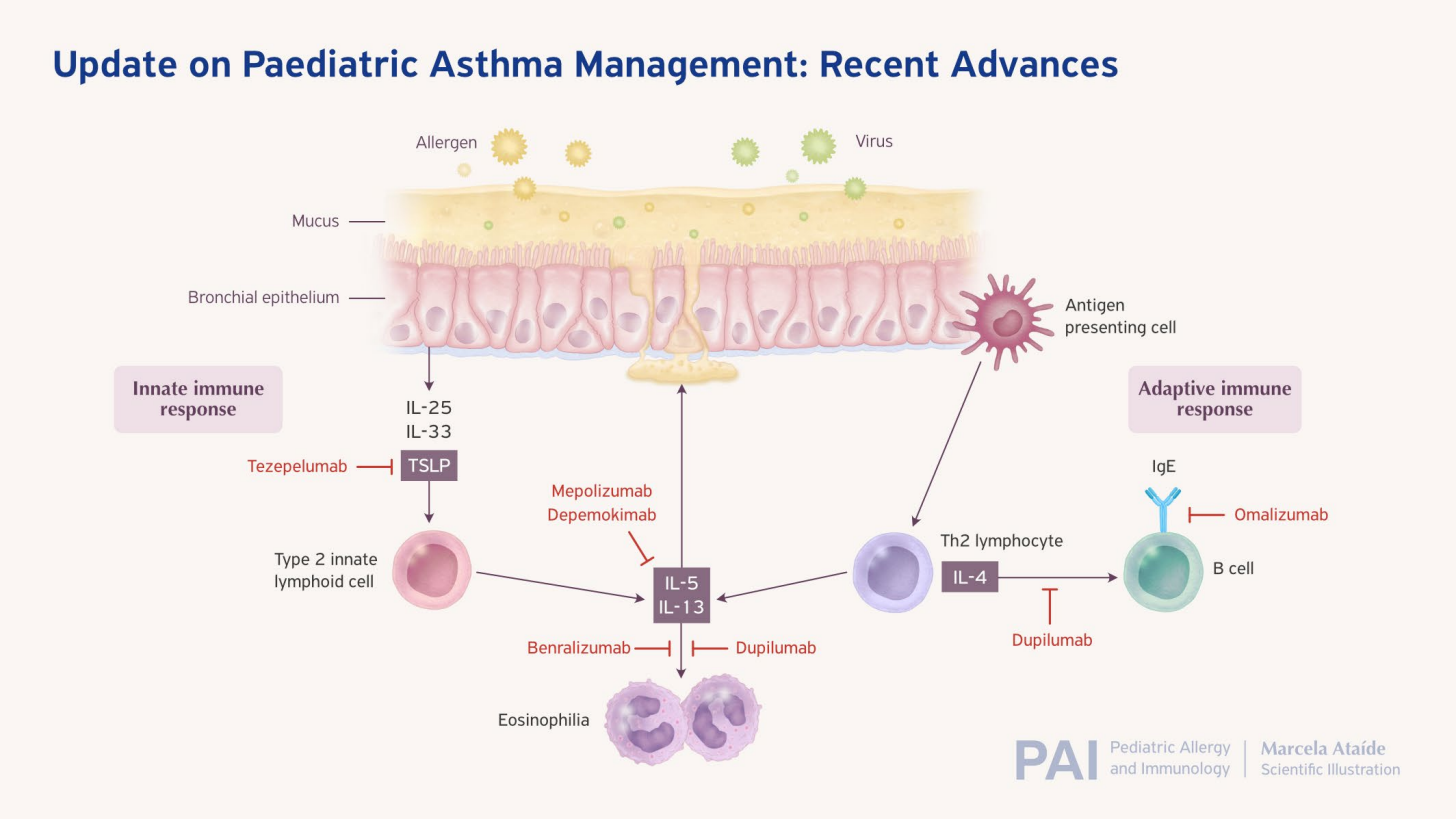

## Graphical abstract

Pathophysiology of asthma: an opportunity for personalized targeted therapeutics.

**Key message**

Recent advances in pediatric asthma management focus on early control of airway inflammation, exacerbation prevention, and personalizing biomarkers to target biologics allowing steroid stewardship and aiming to prevent life‐long loss of lung function.

## 1 INTRODUCTION

Asthma is the commonest chronic respiratory disease affecting children. According to estimates from the Global Burden of Disease Study 2021, there were approximately 126 million prevalent cases and 27 million incident cases of asthma among children globally, with 15,372 (95% uncertainty interval 12,345–19,251) asthma‐related deaths.^1^ Over the past decade, the management of childhood asthma has evolved considerably, shifting from strategies primarily focused on symptom relief to approaches aimed at achieving early control of airway inflammation and preventing exacerbations. The emergence of anti‐inflammatory reliever [AIR] therapies, including inhaled corticosteroid [ICS] containing rescue medications and maintenance‐and‐reliever therapy [MART] using ICS–formoterol, has challenged the traditional reliance on short‐acting β_2_‐agonists [SABA]. These newer strategies offer opportunities to reduce the risk of severe exacerbations in children. In parallel, advances in the understanding of type 2 airway inflammation have led to the development and expanding use of biologic therapies for severe pediatric asthma.^2^ This review highlights recent developments in childhood asthma management and examines their potential impact on long‐term outcomes, focusing on children aged 6–16 years.

## 2 ADVANCES IN MILD–MODERATE PEDIATRIC ASTHMA MANAGEMENT

### 2.1 Anti‐inflammatory reliever therapy

AIR therapy refers to the use of ICS in combination with a rapid‐acting bronchodilator for the relief of acute asthma symptoms. AIR may be delivered through a single combination inhaler containing either ICS and a rapid‐onset long‐acting β_2_‐agonist [LABA] (formoterol), or ICS and SABA in one device, or by administering ICS and SABA via separate inhalers used concurrently.^2^ Details of available formularies and instruction for use of AIR inhalers can be found in the Global Initiative for Asthma [GINA] guidelines and national guidelines.^2^, ^3^

#### 2.1.1 Rationale for AIR

The rationale for AIR therapy in asthma is supported by both physiological mechanisms and clinical evidence. While SABA provide rapid bronchodilatation and relief of airflow obstruction during acute symptoms, they do not address the underlying airway inflammation. In contrast, ICS target the inflammatory component, which is central to asthma pathophysiology. A study of bronchial biopsies and bronchoalveolar lavage [BAL] in asthma patients and healthy controls demonstrated that airway inflammation is present even in mild intermittent asthma. Biopsies showed higher eosinophils and activated T cell infiltration and BAL had higher eosinophil cationic protein in mild asthma compared with healthy individuals.^4^ Untreated ongoing eosinophilic inflammation has the potential to predispose to acute exacerbations, which may become life‐threatening.^5^

The rapid symptomatic relief achieved with SABA, compared with the slower onset of ICS, often leads to overreliance on bronchodilators and suboptimal adherence to ICS. Evidence from large population studies supports the clinical consequences of this imbalance. In an analysis of the Clinical Practice Research Datalink [CPRD] Aurum database involving 90,989 children, the risk of asthma‐related hospital admissions increased in a stepwise manner with higher SABA prescription frequency; children receiving ≥7 prescriptions per year had an incidence rate ratio [IRR] of 4.97 (95% CI 4.06–8.09).^6^ Furthermore, findings from the National Review of Asthma Deaths [NRAD] 2014 showed 58% of individuals who died from asthma had previously been classified as having mild or moderate disease. However, excessive SABA use (≥12 prescriptions annually in 39% of cases) and under‐prescription of preventer therapy were the common contributory features to asthma deaths.^7^ GINA now recommends against SABA‐only treatment. This was introduced in 2019 initially for adolescents and adults, and more recently extended to children aged 6–11 years in 2023.^8^

#### 2.1.2 Anti‐inflammatory reliever therapy alone

When AIR therapy is used as standalone treatment in children with infrequent and intermittent symptoms, it is termed AIR only. AIR only is the recommended GINA Step 1 therapy for all children aged ≥6 years.

Evidence in favor of AIR in adolescents and adults is strong, based on large trials and meta‐analysis.^9^, ^10^, ^11^ A meta‐analysis from 2025 including 27 randomized control trials [RCTs] (*n* = 50,496) showed that ICS‐formoterol (RR 0.65, RD −10.3%) and ICS‐SABA (RR 0.84, RD −4.7%) significantly reduced severe exacerbations vs. SABA alone, with better asthma control and no increase in serious adverse events. ICS‐formoterol also modestly outperformed ICS‐SABA for exacerbation reduction (RR 0.78, 95% CI 0.66–0.92).^10^

The most robust evidence in support of ICS‐formoterol AIR in children comes from the recently published Children's Anti‐Inflammatory Reliever [CARE] study.^12^ It was a 52‐week, open‐label, multicentre, superiority RCT conducted in children aged 5–15 years with mild asthma across 15 sites in New Zealand, comparing as‐needed budesonide–formoterol (50mcg/3mcg) (intervention group) with salbutamol (100 mcg). Three hundred sixty participants were randomized (179 vs. 181), and the annualized asthma attack rate was significantly lower with budesonide–formoterol than salbutamol (0.23 vs. 0.41 events per participant‐year; relative rate 0.55, 95% CI 0.35–0.86; *p* = .012). Effect of intervention was more pronounced for age group 12–15 years, boys and those with high baseline Fractional Exhaled Nitric Oxide [FeNO] (44 ppb and above). Fewer participants in the intervention group experienced at least one attack (17% vs. 32%; OR 0.43, 95% CI 0.24–0.75), and time to first attack was longer in this group (HR 0.48, 95% CI 0.31–0.74). Adverse events were comparable between groups (91% vs. 92%). There was no significant difference in growth velocity (mean difference − 0.35, 95% CI –0.93 to 0.24) and mean (SD) oral corticosteroid [OCS] exposure [18.51 (71.88) mg vs. 26.57 (72.13) mg] in budesonide‐formoterol vs. salbutamol group. Overall, budesonide‐formoterol combination AIR was superior to salbutamol for as needed treatment of mild asthma.^12^

Previous smaller trials in children have consistently demonstrated better outcomes with ICS–SABA compared with SABA‐only therapy in mild asthma. The Treating Children to Prevent Exacerbations of Asthma [TREXA] study, a double‐blind trial conducted in children aged 5–18 years in America showed that rescue therapy with beclomethasone–salbutamol was associated with a lower frequency of exacerbations than salbutamol alone, without the reduction in growth velocity observed in groups receiving daily beclomethasone.^13^ The Asthma Symptom‐based adjustment of Inhaled Steroid Therapy [ASIST] study in African‐American children (aged 6–17 years) showed similar symptom control with as needed ICS‐SABA compared to regular use of ICS and rescue SABA.^14^ Both these studies used separate inhalers for ICS and SABA, used at the same time as the form of AIR therapy.

### 2.2 Maintenance and reliever therapy [MART]

MART consists of a single ICS–formoterol inhaler that is utilized both as a daily controller treatment and as needed for symptom relief.^2^ GINA 2026 guidelines recommend MART with an ICS–formoterol combination as the preferred maintenance treatment for asthma in adolescents (≥12 years) and as one of the preferred options in children aged 6–11 years.^2^ MART offers the advantage of delivering early, proportionate anti‐inflammatory medication (ICS) during symptom worsening, compared with SABA alone. The use of a single inhaler also improves adherence by reducing the need to carry multiple devices. The evidence base in adolescents and adults is robust, demonstrating superiority over alternative strategies. In a 2022 meta‐analysis of pooled individual data in adolescents and adults (*n* = 4863), MART with budesonide–formoterol in poorly controlled asthma was associated with reduced severe exacerbations (requiring OCS for at least 3 days and/or hospitalization) and a longer time to first exacerbation compared with conventional ICS–LABA maintenance plus SABA reliever therapy.^15^

Evidence is limited in the younger population. A 12‐month, double‐blind RCT in 341 children (aged 4–11 years) with uncontrolled asthma compared MART (budesonide–formoterol used for both maintenance and relief) versus fixed‐dose ICS/LABA and higher‐dose ICS regimens. MART group had significantly less exacerbations (14% vs. 38% and 26%), less exacerbations requiring medical intervention (8% vs. 31% and 20%), and prolonged time to first exacerbation compared to fixed dose ICS/LABA or high‐dose ICS. Moreover, MART was associated with improved growth (~1 cm/year) compared to high‐dose ICS.^16^

There are other large ongoing clinical trials of MART in children (START CARE ACTRN12622001217796p, CARE UK ISRCTN65432808) which will provide more definitive evidence for benefit in children. START CARE is a 52 week, multicentre, open‐label, parallel‐group phase III superiority RCT from New Zealand aiming to enroll 400 children aged 5–11 years (1:1) to stepwise budesonide–formoterol reliever‐based therapy (AIR only or MART) versus standard ICS ± LABA plus SABA, with the primary outcome being annualized moderate/severe asthma exacerbation rates.^17^ CARE UK is a UK based phase III, open‐label, parallel‐group RCT aiming to recruit 1352 children aged 6–11 years across multiple centres, allocating them to usual care versus budesonide–formoterol reliever‐based therapy (AIR only or MART) with the primary outcome of annual rates of severe asthma attacks over 52 weeks.^18^ Findings from these studies will consolidate the evidence basis for MART in the pediatric age group.

Clinical trials of AIR and MART in pediatric age group are summarized in Table 1.

**TABLE 1:** Clinical trials of AIR and MART in children.

Study	Population	Intervention (I) and comparator (C)	Follow‐up	Acute exacerbation	Other key findings
Hatter et al., 2025 (CARE)^12^	5–15 years *N* = 382 Mild asthma Australian and New Zealand	I: As‐needed budesonide–formoterol (50mcg/3mcg) C: As‐needed salbutamol (100 mcg)	52 weeks	Asthma attack rate was 0.23 vs 0.41 attacks per patient‐year (*p* = .012), in budesonide–formoterol vs. control.	Longer time to first attack. More participants remained on Step 1 therapy. No significant differences in growth, lung function, symptom control, or adverse events.
Sumino et al., 2020 (ASIST)^14^	6–17 years *N* = 206 Mild asthma African American children in USA	I: Beclomethasone (40 mcg) taken whenever salbutamol was used. C: Daily beclomethasone + as needed salbutamol.	12 months	Exacerbations occurred in 19% of the symptom‐based group and 23% of the daily (*p* = .62) treatment group, with no significant difference	Comparable asthma control and lung function Lower corticosteroid exposure in the intervention group.
Martinez et al., 2011 (TREXA)^13^	5–18 years *N* = 288 Mild persistent asthma USA	I: Combined: daily beclomethasone + rescue beclomethasone/salbutamol (group 1) Daily beclomethasone + rescue salbutamol (group 2) Rescue beclomethasone/ salbutamol only (group 3) C: Placebo plus rescue salbutamol alone (group 4)	44 weeks	Exacerbations requiring oral corticosteroids in group 1,2,3,4 were 31%, 28%, 35% and 49% respectively. Group 3 vs. 4: *p* value −.073	Group 1 and 2 had reduced growth velocity but no difference in group 3 compared with group 4.
Bisgaard et al., 2006^16^	4–11 years *N* = 341 Asthma inadequately controlled on inhaled corticosteroids Europe	I: Budesonide–formoterol (80/4.5 mcg) MART C: Terbutaline rescue with regular budesonide ± formoterol	12 months	Exacerbations requiring medical intervention 0.08 events/patient‐year with MART compared with 0.28 and 0.40 events/patient‐year with regular budesonide‐formoterol and budesonide respectively (*p* < .001).	Prolonged time to first exacerbation in MART Approximately 1 cm greater annual growth in MART

### 2.3 ICS‐formoterol vs. ICS‐SABA

Both ICS–formoterol and ICS–SABA strategies deliver anti‐inflammatory therapy via ICS combined with rapid bronchodilatation. Formoterol has a longer duration of action and has been shown to provide more sustained bronchodilatation and improvement in Forced Expiratory Volume in 1 s [FEV_1_] compared with salbutamol.^19^, ^20^ ICS–formoterol can be used as MART and may improve adherence through single‐inhaler use; however, it is not recommended as a reliever alongside other ICS–LABA regimens due to limited safety evidence.^21^

There are no direct head‐to‐head trials comparing ICS–formoterol and ICS–SABA. Indirect evidence from a network meta‐analysis suggests that ICS–formoterol was associated with a greater reduction in severe exacerbations than ICS–SABA (RR 0.78, 95% CI 0.66–0.92), with similar effects on symptoms and safety.^11^ However, the number of patients studied with ICS–SABA was smaller, and most data were derived from adolescent and adult populations, highlighting the overall lack of high‐quality comparative evidence between these two anti‐inflammatory reliever strategies.

### 2.4 Steroid stewardship

Corticosteroids are the cornerstone and life‐saving treatment for asthma exacerbations and long‐term management. Poor symptom control and repeated exacerbations increase steroid exposure because of the need for frequent OCS and escalation of maintenance therapy to higher doses of ICS. The burden of repeated OCS bursts has been associated with long term adverse effects on bone health, diabetes, and predisposition for infections in adults.^22^, ^23^ ICS use is relatively safe unless used in high doses for a long time.^24^ Additional concerns in children include the effect on height velocity, adrenal suppression and predisposition to infections. Steroid stewardship has been proposed to tackle the overuse of OCS and optimize long‐term inhaled therapy. These include optimisation of ICS, use of AIR to reduce exacerbations, use of appropriate add‐on therapies when indicated, recognition of OCS overuse, and standardization of acute care pathway for appropriate duration of OCS at lowest possible dose.^25^ AIR and MART provide extra dose of ICS when using the reliever hence potentially increasing the ICS exposure. But these therapies have been consistently associated with reduced acute asthma attacks and hence reduce the corticosteroid exposure by preventing OCS bursts promoting steroid stewardship.^11^, ^12^, ^16^

## 3 PEDIATRIC SEVERE ASTHMA: ADVANCES IN USE OF BIOLOGICAL PRODUCTS

### 3.1 Severe asthma

#### 3.1.1 Definition

The European Respiratory Society [ERS] & American Thoracic Society [ATS] guidelines define severe asthma as requiring high‐dose ICS plus a second controller such as LABA (and/or OCS) to maintain control; or uncontrolled asthma despite this therapy.^26^ Uncontrolled pediatric asthma means having either poor symptom control (Asthma Control Test [ACT] score of less than 20) or frequent severe exacerbations (requiring OCS or hospitalization).

The 2026 GINA guidelines classify severe asthma as requiring treatment in the previous year that meets GINA steps 4–5. This would be the equivalent of high‐dose (age‐specific) ICS (Table 2), with LABA and may also include a leukotriene receptor antagonist, theophylline or maintenance OCS for at least 6 months.^2^

**TABLE 2:** Pediatric Asthma age and drug specific high dose inhaled corticosteroids as per 2026 GINA guidelines.^66^

Inhaled corticosteroid	6–11 years old	≥12 years old
Fluticasone propionate	≥200 mcg	≥500 mcg
Beclomethasone dipropionate	≥400 mcg	≥1000 mcg
Budesonide	≥400 mcg	≥800 mcg
Fluticasone Fuorate	N/A	≥200 mcg (DPI only)

*Note*: Doses cover both dry powder inhaler (DPI) and metered dose inhaler (MDI) unless specified.

Abbreviation: mcg, Micrograms.

It is important to remember that all the above refers to poor control on the *prescribed* therapy; there is no confirmation that the therapy has been taken. This is where the distinction between difficult‐to‐treat asthma and true severe asthma becomes important.

#### 3.1.2 Difficult‐to‐treat asthma versus severe asthma

Difficult‐to‐treat asthma has modifiable contributory factors and requires a multi‐disciplinary management model to review diagnosis, adherence & inhaler technique, allergens, environmental exposures and psychosocial factors.^27^ However, it can be challenging to address modifiable factors, even when they are known to be present in a proportion of children. These children are said to have refractory difficult asthma.^28^

There remains a further group, who after optimizing all modifiable factors will still have poor control, and may require further investigations such as bronchoscopy (particularly if infection is suspected) or assessment of steroid responsiveness, and subsequently may progress to biologic therapies.^29^ The children who are eligible for biologics sub‐divide into severe‐treatment resistant asthma (STRA) with poor control despite good adherence and management of all modifiable factors; and refractory difficult asthma.^30^

### 3.2 Biologics for pediatric asthma

#### 3.2.1 Biologic eligibility criteria

**FIGURE 1:**
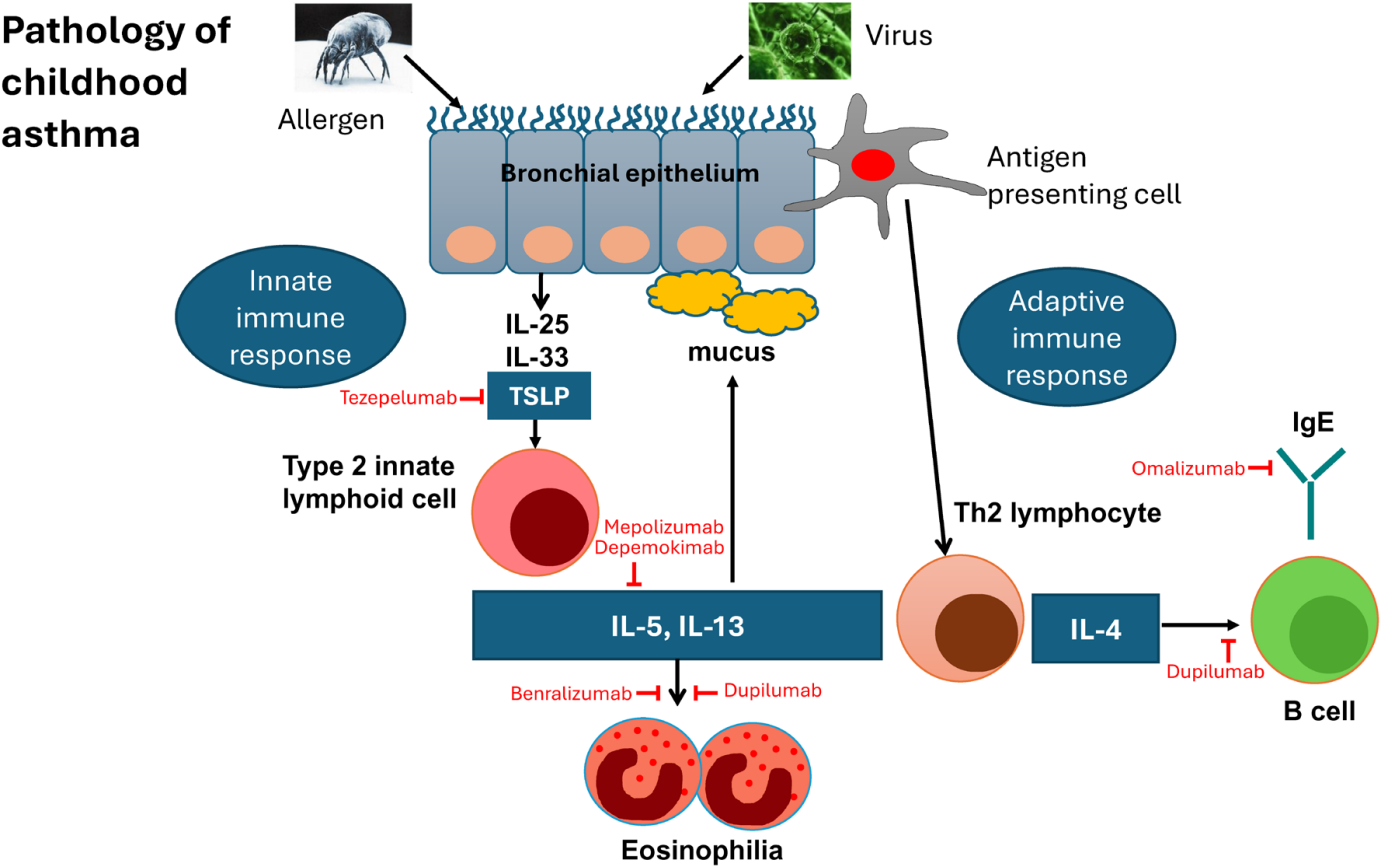
Pediatric asthma pathophysiology and mechanism of action of biologics. IL, Interleukin; TLSP, Thymic Stromal Lymphopoietin; Th2, T helper 2 cell; IgE, Immunoglobulin E.

There are currently six biologic therapies licensed for children with different treatment schedules and recommended biomarkers for use (Table 3). Five are licensed in Europe, and three are licensed for children aged 6 years and above.^31^, ^32^ All are most effective in dampening T helper 2 cell [Th2] inflammation. Their mechanism of action is summarized in Figure 1.

**TABLE 3:** Biological products currently licensed for pediatric severe asthma including biomarker eligibility criteria, dosing and side‐effects.

Biologic Year licensed by NICE [year licensed by EMA] (Year licensed by FDA)	Target	Age	Biomarker eligibility criteria				Treatment (Subcutaneous)		Side effects
			Asthma attacks/last year	OCS courses/last year	Blood eosinophils (cells/μL)	Other criteria	Dose	Weekly interval	
Omalizumab 2013 [2015] (2016)	IgE	6y+	≥4	≥4		IgE 30–1500 IU/mL + aero‐allergen sensitisation	On serum IgE and weight	2–4	Delayed allergic type III reaction, thrombocytopaenia, EGPA, URTIs, Helminthic infections
Mepolizumab 2021 [2018] (2019)	IL‐5	6y+	≥4	≥4	≥300/μL		6–11y 40 mg ≥12y 100 mg	4	Hypersensitivity reactions, Helminthic infections
			≥3	≥3	≥400/μL				
				or Pred‐nisolone 5 mg/day equivalent over 6 m					
Depemokimab Pending [2025] (2025)	IL‐5	12y+	≥2	≥2	≥300/μL in previous 12 m or ≥150/μL at screening.	Medium/High dose ICS for 12 m, plus an additional controller for 3 m. FEV1 <90% predicted or FEV1/FVC <0.8	≥12y 100 mg	26	Higher rate of raised liver function tests thought to be unrelated to drug. One study showed slight raised rate of influenza, one showed decreased rate of influenza.
Benralizumab Not licensed [Not licensed] (2024)	IL‐5 R	6y+	≥2	≥2		Severe (medium/high dose LABA +/− OCS/ preventer) Reduced FEV1	<35 kg 10 mg ≥35 kg 30 mg	4 (1st three doses) then 8	Hypersensitivity reactions, URTIs, Helminthic infections
Dupilumab 2021 [2022] (2021)	IL4‐Rα	6y+	≥4		≥150/μL (or FeNO ≥25 ppb)		6–11y on weight. ≥12y 400 mg then 200 mg	2	Hypersensitivity reactions, ocular side effects, URTIs, EGPA, transient hypereosinophilia, Helminthic infections
Tezepelumab 2023 [2022] (2021)	TSLP	12y+	≥3	≥3 or main‐tenance			210 mg	4	Hypersensitivity reactions, URTIs.

Abbreviations: EGPA, Eosinophilic Granulomatosis with Polyangiitis; EMA, European Medicines Agency; FDA, Food and Drug Administration; FeNO, Fractional Exhaled Nitric Oxide; FEV1, Forced Expiratory Volume in 1 s; IgE, Immunoglobulin E; IL4‐Rα, Interleukin 4 Receptor alpha; IL‐5, Interleukin 5; IL‐5R, Interleukin 5 receptor; IU, International Units; mL, milliliter; NICE, National Institute for Health and Care Excellence; OCS, Oral Corticosteroids; TLSP, Thymic Stromal Lymphopoietin; URTI, Upper Respiratory Tract Infection; μL, microlitre.

All biologics are humanized monoclonal antibodies delivered by subcutaneous injection 2–26 weekly, initially in hospital to ensure safety and then often at home with regular monitoring. GINA guidelines recommend reviewing response within 3–4 months to assess whether to continue biologic agents, and subsequently, every 3–6 months aiming for a minimum of 12 months.^2^

#### 3.2.2 Biomarkers and composite measures of biologic efficacy

Pediatric biologic efficacy data was initially extrapolated from predominantly adult studies. There are currently no published head‐to‐head trials of the different biologics in children. The main outcome of biologic efficacy in all published trials is the annualized asthma exacerbation rate [AER]. There are currently no studies that have published childhood‐specific biomarkers, although data on blood eosinophils, FeNO, symptom control, FEV_1_, and Quality of Life [QoL] have been reported (Table 4). Most of these reported biomarkers were not adequately powered as pre‐planned secondary outcomes, and none have been used as the primary outcome in any trial, limiting implementation in real‐world settings. The use of a composite score which incorporates exacerbations, lung function, maintenance OCS, symptom score, and quality of life (such as the CompOsite iNdex for Response in asthMa [CONFiRM] score) to understand the response to biologics in a real‐world setting is crucial.^33^

**TABLE 4:** Published pediatric biologic biomarker data. Based on Table 3 from "How to monitor response to biologics in children with severe asthma".^73^

Biologic	AER reduction versus placebo	FEV_1_	Blood eosinophils	FeNO	Asthma control tests	QoL
Omalizumab	38.5% versus 18.2%^67^ 43% reduction^68^ 48.8% versus 30.3%^69^ OR 0.48 (over 90 days)^62^	62, 67, 69			cACT 0.78 increase^69^	GETE: Excellent: 31.5% versus placebo: 16.3%^67^
					ACT^62^, ^67^, ^69^	^68^
Mepolizumab	27% reduction^41^	41, 70	−299 cells/μL^41^ 87.1% reduction^70^ −289 cells/μL^71^	^41^	70, 71	^41^
	69% reduction^71^					
Depemokimab	Rate ratio 0.46^44^ (*n* = 762, 20 12–17 years)		82%–83% reduction^44^		^44^	^44^
Benralizumab	Low dose: 45.9% reduction High dose: 53.6% reduction^42^	Low dose: + 0.068L High dose: + 0.043L^42^	Low dose: −434 cells/μL High dose: −454 cells/μL^42^		ACQ‐1A Low dose: −0.19, High dose: −0.58^42^	
Dupilumab	59.3% reduction^45^ 200 mg: 46% reduction^47^ 300 mg: 13% increase^47^ 59% reduction^46^	200 mg: +0.37 L 300 mg: +0.27L^47^ + 5.2% in % predicted FEV1 versus placebo^45^ +0.22L^46^ +10.82% in % predicted FEV1 (comorbid atopic dermatitis/rhinitis)^50^	Transient blood eosinophilia: 5.9% versus 0.7% on placebo^45^, ^47^	Th2 group: −17.84 ppb, Blood eosinophils ≥300 cells/μL group −20.59 ppb, versus placebo at 12 weeks^45^	ACQ 7–1.33 versus −1 for placebo^45^	^47^
				At 52 weeks^45^, ^47^	^47^	
Tezepelumab	60% reduction^72^ (*n* = 951, 82 12–17 years)	+0.22L versus +0.09L (placebo)^72^	−0.13 cells/μL versus placebo^72^	Baseline ≥50 ppb: −57.8% Baseline 25–49 ppb: −42.3%^72^	ACQ‐6 −0.33 versus placebo^72^	AQLQ +0.33 versus placebo^72^

*Note*: White: significant data for that biomarker. Light gray: data is non‐significant or not powered for that biomarker. Dark gray: no evidence published on that biomarker.

Abbreviations: ACQ, Asthma Control Questionnaire; ACT, Asthma Control Test; AER, Annualized asthma Exacerbation Rate; AQLQ, Asthma Quality of Life Questionnaire; cACT, Childhood Asthma Control Test; FeNO, Fractional Exhaled Nitric Oxide; FEV_1_, Forced Expiratory Volume in 1 s; GETE, Global Evaluation of Treatment Effectiveness; ppb, parts per billion; QoL, Quality of life; v, versus.

### 3.3 Factors that may help choose the correct biologic for each child

#### 3.3.1 Omalizumab reduces co‐morbid food allergy hospital attendances

Omalizumab targets free serum IgE, forming complexes so it cannot bind to FcERI receptors. This reduces IgE mediated inflammatory responses to allergen exposure (Figure 1) and augments antiviral responses.^34^ As the first biologic licensed in children, it has the largest evidence base and is licensed for patients with serum IgE 30–1500 IU/mL. A meta‐analysis of the main four pediatric RCTs showed omalizumab was effective in decreasing the rate of asthma exacerbations compared to placebo (OR 0.45) and reducing the required dose of ICS by −108 micrograms of budesonide equivalent/day.^35^ A meta‐analysis including three pediatric RCTs found patients receiving omalizumab had a lower incidence of severe adverse events (OR 0.36) (although this included asthma attacks).^36^ For young people on omalizumab ≥12 years, those with elevated FeNO and blood eosinophils had better outcomes.^37^

OUtMATCH demonstrated omalizumab increases the reaction threshold for common food allergens to levels uncommon in accidental exposure.^38^ Comorbid asthma increases the risk of severe bronchospasm and anaphylaxis in food allergy. A healthcare resource utilization study of 523 patients aged ≥6 years who initiated omalizumab demonstrated a significant reduction in the proportion of patients attending the emergency department for co‐morbid asthma and food allergy (baseline 43.8%, vs. 28.5% on omalizumab). This suggests an anti‐IgE biologic may be the optimal choice for children with both severe asthma and food allergy if the patient's serum IgE allows.^39^

#### 3.3.2 Mepolizumab requires targeting to predominant eosinophilic disease

Mepolizumab targets interleukin 5 (IL‐5), preventing it binding to interleukin‐5 receptor alpha (IL‐5Rα), reducing eosinophilic inflammation (Figure 1). A review written by the manufacturers of mepolizumab of the 34 young people (aged 12–17 years) recruited across all four Phase III RCTs suggested that whilst all outcomes were non‐significant in this sub‐group, the direction of trend favored mepolizumab.^40^ However, the MUPPITS‐2 trial of 290 6–17 year‐olds with severe eosinophilic asthma found mepolizumab reduced the AER to 0.96 compared to 1.3 in the placebo group.^41^ This rate reduction (27%) was only half that seen in adult studies. This may be due to the socioeconomic demographics of the intervention population placing them at higher risk for adverse environmental exposures. However, greater gene expression in nasal lavage of non‐eosinophilic inflammatory pathways such as neutrophil chemotaxis and type 1 Interferon regulation (linked with viral and pollution triggered exacerbations) and those related to remodeling (epithelial growth factor receptor) were related to a higher AER in the mepolizumab group compared to placebo. This suggests only a sub‐group of children with severe asthma will respond to mepolizumab, and we need reliable biomarkers of response for children.^41^ A further limitation is mepolizumab's dosage is determined by age, not weight, meaning a large eleven‐year‐old receives the same dose as a small six‐year‐old; this is likely to impact efficacy.

#### 3.3.3 Benralizumab is only licensed for children in certain countries

Benralizumab targets the IL‐5Rα (Figure 1), however, it is less well tolerated and not currently licensed in Europe for children.^42^ The TATE study investigating the pharmacokinetics of Benralizumab in 28 children aged 6–11 years receiving weight‐based dosing (Table 3) reported pre‐bronchodilator FEV_1_ change from baseline. The lower dose group had no change in FEV1 by week 48; the higher dose group had increases ranging from 0.043 L to 0.425 L throughout the 48 week period (with a decrease of −0.119 L at week 16). This was a very small study; FEV_1_ was not a primary outcome, and the majority of error bars crossed zero.^42^ Dominica (NCT05692180) is an ongoing multicentre (including European sites), randomized, double‐blind, placebo‐controlled, Phase III study investigating time to first asthma exacerbation in two hundred 6–17‐year‐olds with severe eosinophilic asthma.^43^

#### 3.3.4 Depemokimab is given 6 monthly but pediatric efficacy data are needed

Depemokimab is an ultra‐long‐acting monoclonal antibody that targets interleukin‐5, enabling 6‐monthly dosing. This makes it attractive for the pediatric population. It is currently licensed in the UK by the EMA and FDA ≥12 years. National Institute for Health and Care Excellence [NICE] cost‐effectiveness assessment is ongoing.^31^ However, the data for efficacy are from adults, with only 30 young people ≥12 years included in the licensing study.^44^

SWIFT‐1 and SWIFT‐2 included 762 asthmatics ≥12 years on regular medium/high‐dose ICS for 12 months plus an additional controller for 3 months, with ≥2 asthma exacerbations requiring OCS in the last year. Required biomarkers were blood eosinophils ≥300/μL in the previous year or ≥150/μL at screening and FEV_1_ <80% in adults or <90% in young people, or FEV_1_/FVC ratio <0.8 in young people. They were randomized to two subcutaneous depemokimab or placebo injections at week 0 and 26 and followed up for 1 year. The AER was significantly lower on depemokimab, 0.51 compared to 1.11 on placebo, with no significant increase in adverse events.^44^

SWIFT‐1 only included 8 young people, 3 of whom received depemokimab, all on medium‐dose ICS, so did not meet severe asthma criteria. SWIFT‐2 included 22 young people, 12 received depemokimab, 9 on high‐dose ICS and met severe asthma criteria. There were no sub‐group analyses of outcomes for young people. No patients had anaphylaxis or hypersensitivity reactions. Efficacy data for depemokimab is needed for children to understand its role in the management of severe pediatric asthma.

#### 3.3.5 Dupilumab increases lung function & improves atopic dermatitis

Dupilumab targets IL‐4 receptor alpha [IL‐4Rα] and blocks IL‐4 and IL‐13 which are involved at multiple steps of type‐2 mediated inflammation. NICE regulation in the UK means it is only available to children who have either not responded to or are ineligible for mepolizumab. Prescribing and pricing pathways in Ireland, Nordic and Eastern European countries require cheaper existing biologics to be used first or demonstration that they are clinically inappropriate. There are robust data showing efficacy of dupilumab in children with severe asthma; however, all pediatric trials to date have been done in children aged 6–11 years. There was a greater reduction in the AER on dupilumab in both pediatric RCTs (VOYAGE 0.42, 1.15 on placebo,^45^ VENTURE 0.65, 1.60 on placebo^46^) compared to that of mepolizumab seen in MUPPITS‐2 above.

Dupilumab is the only pediatric biologic to have multiple studies documenting a significant increase in lung function in young people. In addition to a reduction in AER, VOYAGE demonstrated in 408 children aged 6–11 years with uncontrolled moderate/severe asthma and blood eosinophils ≥150 cells/μL or FeNO ≥20 ppb a significant increase in FEV_1_% predicted of 10.5% with dupilumab versus 5.3% with placebo.^45^

A post‐hoc analysis by the manufacturers of the 107 adolescents aged 12–17 years involved in QUEST comparing dupilumab to placebo demonstrated the greatest published increase in lung function seen in young people on biologics. Dupilumab significantly increased pre‐bronchodilator FEV_1_ versus the placebo group after 12 weeks by 0.37 L in the 200 mg group, and 0.27 L in the 300 mg group. The increase in pre‐bronchodilator FEV_1_ was even higher (0.43 L) in those with blood eosinophils of ≥150 cells/microlitre, or FeNO ≥20 ppb (eligibility criteria is ≥25 ppb).^47^

Dupilumab is also licensed for the treatment of severe atopic dermatitis in children.^48^, ^49^ A study of 25 children aged 6–13 years with moderate/severe asthma and co‐morbid atopic dermatitis or allergic rhinitis demonstrated a significant increase in mean FEV_1_% predicted from 82.03% at baseline to 92.85% at week 24 with significant decreases in atopic dermatitis and allergic rhinitis scores.^50^ This suggests dupilumab would be the biologic of choice for children with atopic co‐morbidities.

A systematic review of six studies of dupilumab in children with moderate to severe asthma used narrative synthesis to conclude dupilumab reduced the AER by 59% (65% if blood eosinophils ≥300 cells/μL, 69% if FeNO ≥50 ppb) with an improvement in pre‐bronchodilator FEV_1_ of 0.15–0.3 L. Improvements in asthma control, QoL, FeNO, blood eosinophils and IgE were noted.^51^

Significantly more young people were able to wean or cease glucocorticoid use on dupilumab compared to placebo.^46^ Dupilumab can be associated with blood hypereosinophilia as an adverse effect (although this is usually self‐resolving), mild ocular side effects, and an increased rate of upper respiratory tract infections.^52^ LIBERTY ASTHMA TREKIDS is recruiting ninety atopic 2–5 year‐olds with recurrent severe preschool wheeze uncontrolled on ICS to assess the efficacy and safety of dupilumab in preschoolers.^53^

#### 3.3.6 Tezepelumab does not require type‐2 eligibility biomarkers; but pediatric efficacy data are needed

Tezepelumab targets thymic stromal lymphopoietin [TLSP] involved not only in type‐2 mediated but also type‐1 inflammation. Tezepelumab is open to young people ≥12 years who do not have biomarkers for type‐2 mediated asthma as well as those who do. There are no pediatric‐specific studies, but NAVIGATOR did report a post‐hoc, unpowered analysis of AER for 82 12–17‐year‐olds showing a reduction to 0.90 on tezepelumab compared to 1.73 on placebo.^54^

The DESTINATION^55^ study analyzed the safety data from the two main Phase III trials (NAVIGATOR,^54^ SOURCE^56^) and found a decrease in adverse events on tezepelumab compared to placebo, noting the majority of participants were adults not young people.^55^ HORIZON (NCT06023589) is an ongoing phase III multicentre, double‐blind RCT comparing the AER in 231 5–11 year‐olds on tezepelumab to placebo.

### 3.4 Choosing the right biologic for each patient

**FIGURE 2:**
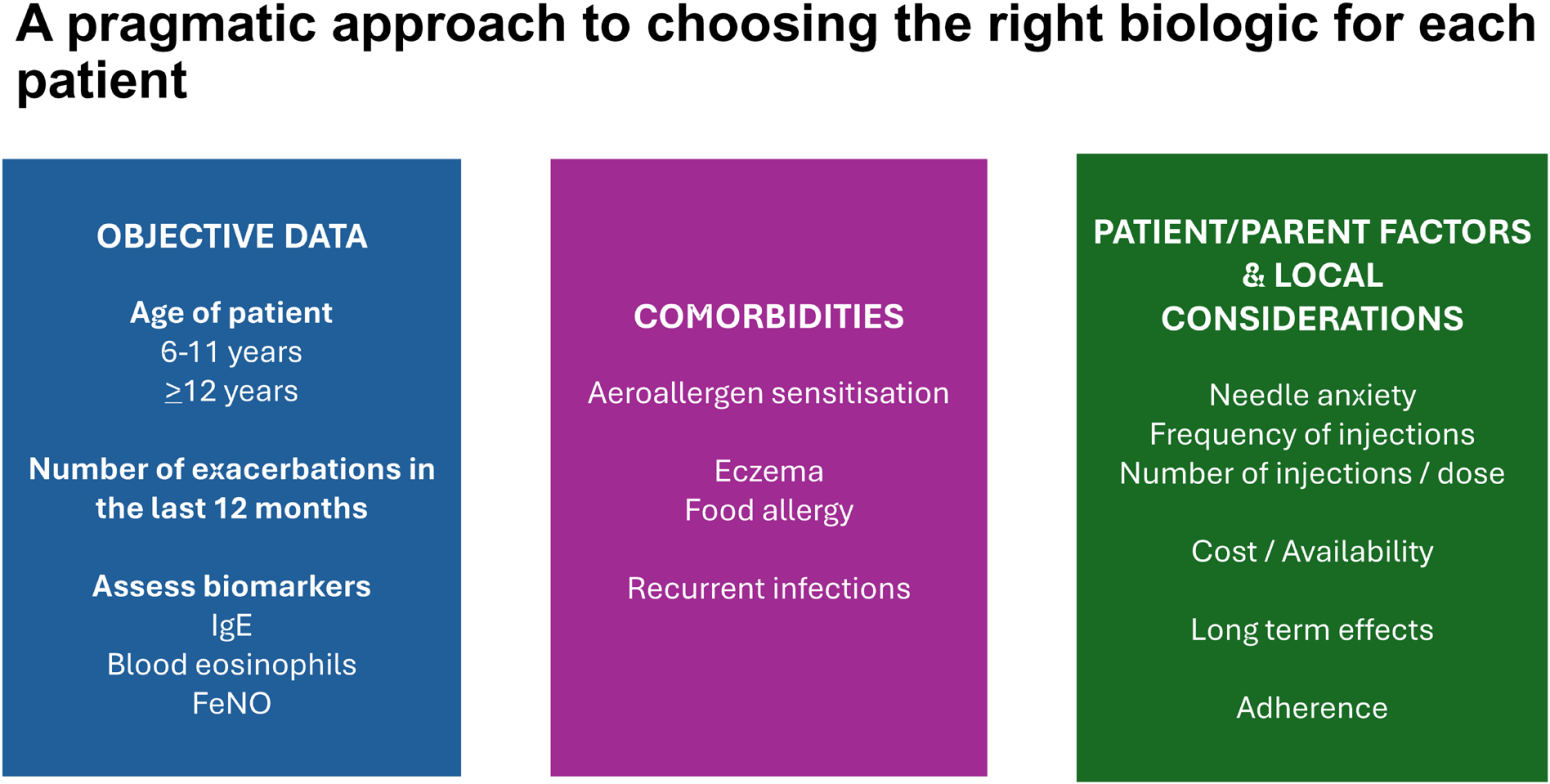
A pragmatic approach to choosing the right biological product for each pediatric patient with severe asthma. IgE, Immunoglobulin E; FeNO, Fractional Exhaled Nitric Oxide.

In addition to the eligibility criteria (Table 3) and the characteristics of each biologic outlined above, each patient requires a personalized approach considering their co‐morbidities, psychosocial factors, and healthcare resource utilization. A pragmatic approach is suggested in Figure 2.

### 3.5 Future considerations in the use of biologics for children

#### 3.5.1 Pediatric biomarkers

For children eligible for multiple biologics according to their biomarkers, we currently lack clear evidence of optimal biomarkers that predict response to each biologic in the pediatric population to guide choice of agent. Eligibility criteria are currently based on adult values for blood eosinophils, FeNO, and serum IgE. However, children may have elevated IgE due to other pathology; blood eosinophils vary by age, environmental factors, and corticosteroid use, and may not correlate with airway eosinophilia.^57^ Currently, dosing determined by weight is only used for omalizumab, which means there may be variation in efficacy for the other biologics in children.

#### 3.5.2 Direct comparisons

The TREAT trial, a multicentre RCT of 500 children aged 6–17 years with severe asthma comparing omalizumab against mepolizumab for 1 year with biomarker independent allocation, is due to complete in 2027.^58^

NIMBLE was a multicentre RCT of 1687 participants ≥12 years with severe asthma on mepolizumab or benralizumab for at least 1 year who were randomized to 6 monthly depemokimab or current treatment. The primary outcome of AER did not meet non‐inferiority criteria. However, the pre‐powered sub‐group analysis of patients switching from mepolizumab to depemokimab did, without a significant rise in blood eosinophils. This is relevant as mepolizumab but not benralizumab is licensed for children in Europe. However, the number of 12–17‐year‐olds in the NIMBLE study was not reported, and a subgroup analysis of young people was not conducted. The mean participant age was 58, making it harder to extrapolate to the pediatric population.^59^

The choice of formulations will widen as omalizumab is now off patent opening the market to biosimilars with a concurrent price reduction.^60^

#### 3.5.3 Biologics to achieve secondary prevention and reduce seasonal peaks in exacerbations

Biologics are being investigated as disease modifying agents and for interval use. The PARK trial, a multicentre RCT assessing whether omalizumab given for 2 years to pre‐schoolers aged 2–3 years at high risk for asthma can prevent the progression to active asthma, is due to complete in 2028.^61^ Similarly LIBERTY ASTHMA TREKIDS is assessing the efficacy and safety of dupilumab in atopic recurrent severe preschool wheezers.^53^

A 4‐month course of pre‐seasonal omalizumab starting 4–6 weeks before the September return to school demonstrated a reduction in asthma exacerbations although not all participants had severe asthma and an increase to maximal ICS had a similar outcome.^62^ However, the advantages of ensuring adherence and steroid stewardship both make this an attractive use in children. In adults with asthma and Chronic Obstructive Pulmonary Disease [COPD], a single dose benralizumab given during an acute exacerbation significantly increased time to the next exacerbation as compared to prednisolone alone in a multicentre RCT.^63^ A phase II RCT is currently investigating rademikibart (targets IL‐4Rα blocking IL‐4/IL‐13) as an add‐on treatment for acute exacerbations in adolescents and adults with type 2 asthma.^64^, ^65^

Unanswered questions and future areas for research in the use of biologics in children (Table 5).

**TABLE 5:** Future directions for biologic products in pediatric asthma.

Areas of unmet need requiring clinical trials and real‐world data
Are the criteria for starting biologics in children too restrictive (number of exacerbations/ICS dose)? ○ Are we starting biologics too late? ○ Risking early loss of lung function? Do we have the correct cut‐off values for biomarkers for children? ○ Blood eosinophils ○ IgE ○ FeNO Should we use the CONFiRM^33^ criteria to determine response? When/How should we switch biologics if they are ineffective? How long should treatment with a biologic continue (once effective) in children? ○ Many children aged 6–11 years improve over time (especially around puberty and males) When/How should we stop biologics?

## 4 CONCLUSION

Contemporary approaches to childhood asthma management increasingly emphasize early control of airway inflammation and the need for childhood‐tailored application of biologics for severe disease. These advances are enabling more personalized treatment strategies that improve disease control, decrease the risk of exacerbations, and critically, may help to prevent the irreversible loss in lung function in early life that may continue from childhood to adulthood and result in COPD. However, further studies are required to identify the patients most likely to benefit from these interventions, enhance equitable access to emerging therapies, and strengthen the implementation of precision medicine in pediatric asthma care.

## AUTHOR CONTRIBUTIONS

**Nitin Dhochak:** Writing – original draft; writing – review and editing; visualization. **Polly F. M. Robinson:** Writing – original draft; writing – review and editing; visualization. **Sejal Saglani:** Conceptualization; visualization; writing – review and editing; supervision.

## FUNDING INFORMATION

Nitin Dhochak, Polly FM Robinson, and Sejal Saglani declare no financial support.

## CONFLICT OF INTEREST STATEMENT

Nitin Dhochak, Polly FM Robinson, and Sejal Saglani declare no conflicts of interest.

## Data Availability

Data sharing not applicable to this article as no datasets were generated or analyzed during the current study.
